# Distribution, cellular localization, and colocalization of several peptide neurotransmitters in the central nervous system of *Aplysia*

**DOI:** 10.1101/lm.053758.123

**Published:** 2023

**Authors:** Robert D. Hawkins, Lennart Brodin, Elvar Theodorsson, Ákos Végvári, Eric R. Kandel, Tomas Hokfelt

**Affiliations:** 1 Department of Neuroscience, Columbia University, New York, New York 10032, USA; 2 New York State Psychiatric Institute, New York, New York 10032, USA; 3 Department of Neuroscience, Karolinska Institutet, Stockholm S-17177, Sweden; 4 Department of Biomedical and Clinical Sciences, Division of Clinical Chemistry and Pharmacology, Linköping University, Linköping S-58185, Sweden; 5 Department of Medical Biochemistry and Biophysics, Karolinska Institutet, Stockholm S-17177, Sweden; 6 Howard Hughes Medical Institute, New York, New York 10032, USA

## Abstract

Neuropeptides are widely used as neurotransmitters in vertebrates and invertebrates. In vertebrates, a detailed understanding of their functions as transmitters has been hampered by the complexity of the nervous system. The marine mollusk *Aplysia*, with a simpler nervous system and many large, identified neurons, presents several advantages for addressing this question and has been used to examine the roles of tens of peptides in behavior. To screen for other peptides that might also play roles in behavior, we observed immunoreactivity in individual neurons in the central nervous system of adult *Aplysia* with antisera raised against the *Aplysia* peptide FMRFamide and two mammalian peptides that are also found in *Aplysia*, cholecystokinin (CCK) and neuropeptide Y (NPY), as well as serotonin (5HT). In addition, we observed staining of individual neurons with antisera raised against mammalian somatostatin (SOM) and peptide histidine isoleucine (PHI). However, genomic analysis has shown that these two peptides are not expressed in the *Aplysia* nervous system, and we have therefore labeled the unknown peptides stained by these two antibodies as X_SOM_ and X_PHI_. There was an area at the anterior end of the cerebral ganglion that had staining by antisera raised against many different transmitters, suggesting that this may be a modulatory region of the nervous system. There was also staining for X_SOM_ and, in some cases, FMRFamide in the bag cell cluster of the abdominal ganglion. In addition, these and other studies have revealed a fairly high degree of colocalization of different neuropeptides in individual neurons, suggesting that the peptides do not just act independently but can also interact in different combinations to produce complex functions. The simple nervous system of *Aplysia* is advantageous for further testing these ideas.

A number of peptides that were originally found to have hormonal functions in the body have also been found to act as neurotransmitters in the nervous systems of both vertebrates and invertebrates. In vertebrates, a detailed understanding of their functions as transmitters has been hampered by the complexity of the nervous system. Invertebrates such as *Aplysia*, *Drosophila* (Nässel and Zandawala 2019), *Caenorhabditis elegans* (Li et al. 1999), and crabs (Nusbaum et al. 2017) with simpler nervous systems and in some cases large, identified neurons are advantageous for addressing this question. For example, studies in *Aplysia* have shown that the neuropeptide Phe–Met–Arg–Phe–NH2 (FMRFamide) is expressed in an individual identified neuron that plays an important role in synaptic and behavioral inhibition (Small et al. 1992). FMRFamide and several other peptides have also been associated with regulation of feeding behavior (Lloyd et al. 1987; Ono 1989; Cropper et al. 1991; Brezina et al. 1995; Vilim et al. 2000; Chan-Andersen et al. 2022) and egg laying (Strumwasser et al. 1980; Sossin et al. 1990; Rajpara et al. 1992) in *Aplysia*.

To look for other peptides that might also play roles in behavior, we examined the distribution and cellular localization of immunoreactivity using primary antisera raised against several additional mammalian peptides, some of which had previously been described in the *Aplysia* nervous system, which expresses several hundred genes of this type (Chan-Andersen et al. 2022). We observed immunoreactivity in individual neurons in the central nervous system of adult *Aplysia* with antisera raised against the *Aplysia* peptide FMRFamide and two mammalian peptides that are also found in *Aplysia*, cholecystokinin (CCK) and neuropeptide Y (NPY), as well as serotonin (5HT). In addition, we observed staining of individual neurons with antisera raised against mammalian somatostatin (SOM) and peptide histidine isoleucine (PHI). However, genomic and transcriptomic analyses have shown that these two peptides are not expressed in the *Aplysia* nervous system, and evolutionary analyses suggest that they are only found in vertebrates (Jékely 2013; Jiang et al. 2022; Orvis et al. 2022). We therefore say that there is staining for X_SOM_ or X_PHI_, where X is an unknown *Aplysia* peptide and the subscript indicates that it is recognized by antisera raised against mammalian SOM or PHI. It remains to be seen whether these two *Aplysia* peptides are more or less distant relatives of the mammalian peptides. However, these results suggest that these or immunogenically similar peptides are candidates to play modulatory roles in additional aspects of behavior.

Interestingly, these and other studies have revealed a fairly high degree of colocalization of labeling with antisera raised against different neuropeptides in individual neurons, suggesting that the peptides do not just act independently but rather are coreleased in different combinations and thus have different neuronal functions dependent on the combinations of receptors and G proteins that they activate (Lloyd et al. 1987; Cropper et al. 1991; Brezina et al. 1995; Vilim et al. 2000; Chan-Andersen et al. 2022). Thus, for example, buccal motor neurons corelease a number of peptides that can have different effects individually and in combination (Cropper et al. 1991; Brezina et al. 1995; Vilim et al. 2000; Chan-Andersen et al. 2022). The simple nervous system of *Aplysia* is advantageous for further testing the roles of peptides and their combinations in behavior.

## Results

In a preliminary screen, we performed immunofluorescence histochemistry with primary antisera raised against 13 different mammalian neuropeptides, including CCK, SOM, PHI, NPY, neuropeptide K (NPK), substance P (SP), thyrotropin-releasing hormone (TRH), peptide tyrosine tyrosine (PYY), bradykinin potentiating peptide (BPP), enkephalin (Enk), calcitonin gene-related peptide (CGRP), galanin (Gal), and neurotensin (NT), plus *Aplysia* FMRFamide and the monoamine neurotransmitter serotonin (5-hydroxytryptamine [5HT]) (Table 1). We and others had previously studied all of the mammalian neuropeptides with immunohistochemistry in rats (e.g., Eriksdotter-Nilsson et al. 1987; Ceccatelli et al. 1989; Lindh et al. 1989; Hökfelt 1991). We were now interested in examining whether they are also expressed or have relatives in *Aplysia*, where we subsequently could more easily examine their functions in plasticity and behavior and possibly compare those with mammals. Of these, 11 peptide antisera produced staining of cell bodies and fibers in the five major ganglia of the *Aplysia* central nervous system (abdominal, pedal, pleural, cerebral, and buccal ganglia) and three were negative. Based on the results of this initial screen, for the remaining experiments we focused on antisera raised against the *Aplysia* peptide FMRFamide and the mammalian peptides CCK, NPY, SOM, and PHI as well as 5HT.

**Table 1.**
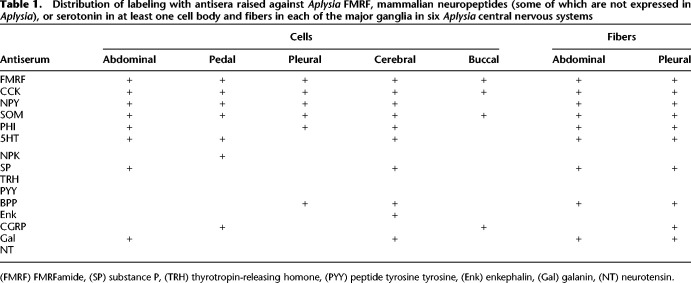
Distribution of labeling with antisera raised against *Aplysia* FMRF, mammalian neuropeptides (some of which are not expressed in *Aplysia*), or serotonin in at least one cell body and fibers in each of the major ganglia in six *Aplysia* central nervous systems

The antisera were applied on serial cryostat sections of central nervous systems from two adult *Aplysia* in the preliminary screen and four more in the remaining experiments. The large size of many neurons (often >100 µm) makes it possible for a single neuron to be identified in several consecutive sections (10–12 µm thick) that then can be stained with different antibodies. This approach avoids problems associated with double or triple staining by applying two or three antibodies on the same section. However, it may miss some positive neurons because it only stains alternating sections for each transmitter. This was particularly an issue in the preliminary screen, where we stained only about every 15th section (out of 50–100 per nervous system) for a given transmitter, but it was also an issue in the remaining experiments, where we stained every sixth or seventh section for a given transmitter. Thus, there was a tradeoff between the number of transmitters we mapped and the completeness of the maps for each one in the preliminary screen and the subsequent experiments.

**Figure 1.**
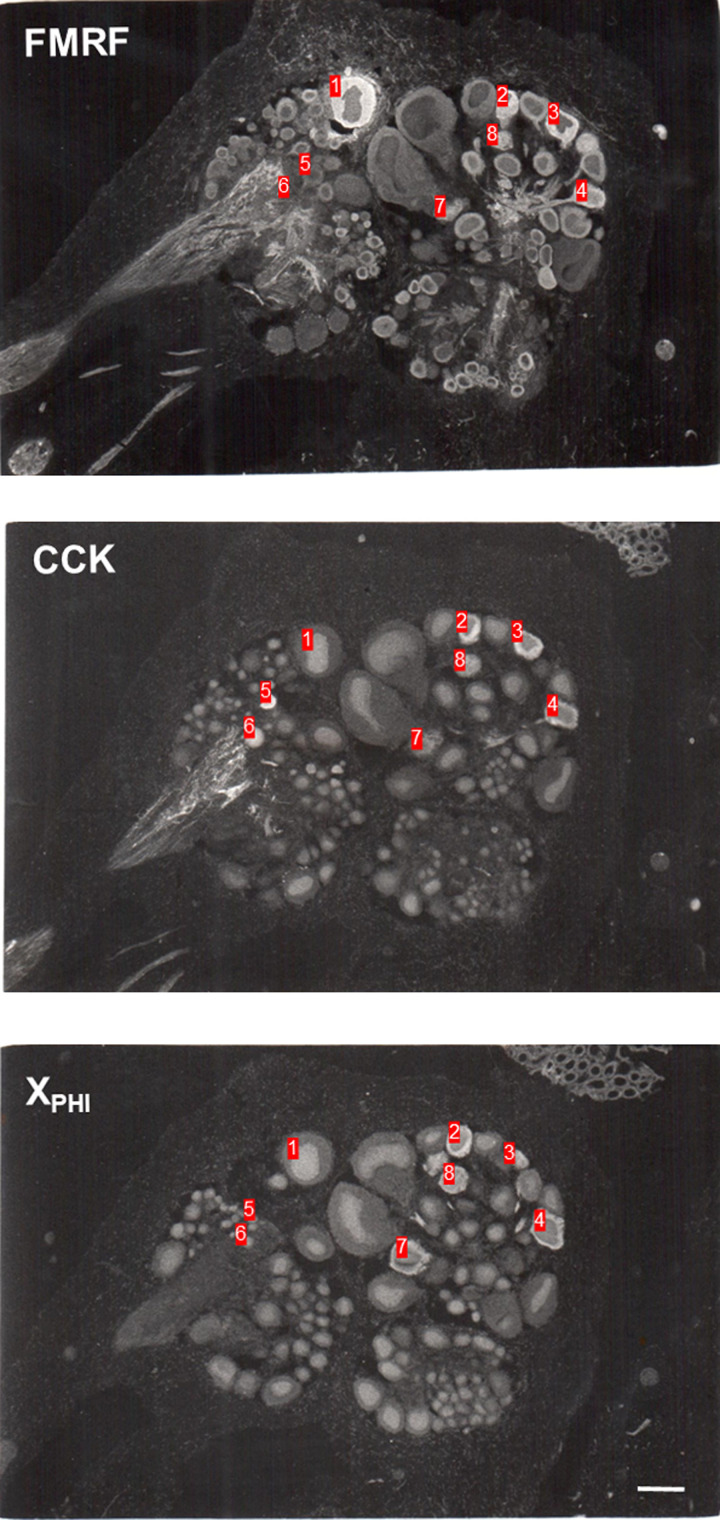
Examples of staining with antisera raised against *Aplysia* Phe–Met–Arg–Phe–NH2 (FMRFamide [FMRF]) and mammalian cholecystokinin (CCK) and peptide histidine isoleucine (PHI) in the cytoplasm of some cell bodies and also fibers in adjacent cryostat sections of the abdominal ganglion of *Aplysia.* The anti-PHI antiserum stains an unknown peptide, X_PHI_. The numbers indicate individual neurons that stained with antisera raised against FMRFamide alone (1), CCK alone (5 and 6), FMRFamide and PHI (7), or all three (2, 3, 4, and 8). Scale bar, 200 µm.

**Figure 2.**
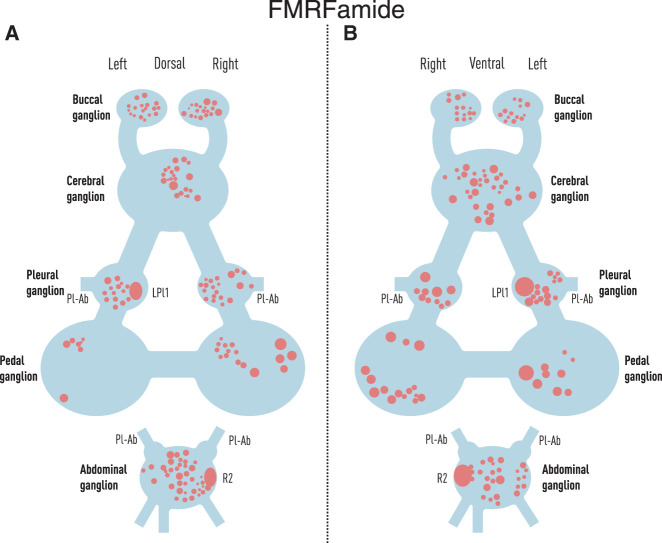
The approximate location and size of neurons that stained with antisera raised against FMRFamide in the central nervous systems of adult *Aplysia*, reconstructed from serial sections of two nervous systems. (*A*) Dorsal view of the dorsal half of the nervous system. (*B*) Ventral view of the ventral half of the nervous system. (Pl-Ab) Pleural–abdominal connective.

Figure 1 shows examples of staining of adjacent sections of the abdominal ganglion after incubation with antiserum raised against FMRFamide, CCK, or mammalian PHI. The immunoreactivity is seen in the cytoplasm of some cell bodies and also in fibers. Figure 2, A and B, shows a map of cell bodies that stained with antisera raised against FMRFamide reconstructed from every sixth or seventh section from two central nervous systems, with some additional sections stained with anti-FMRFamide antiserum to construct a better map of that transmitter. There are FMRFamide-positive cells in all of the ganglia, including the giant identified neurons R2 and LPl1, in agreement with previous studies (Brown et al. 1985; Small et al. 1992). The position, size, and number of the cells are also generally similar to those seen previously in whole mounts of juvenile *Aplysia* (Small et al. 1992). Comparing the total number of cells in juveniles versus adults is confounded with the methods used (whole mounts vs. sections, which may miss some cells) (see above). However, we observed a difference in the relative distribution of cells that stained with anti-FMRFamide antiserum in the different ganglia (χ^2^ = 28.0 with 4 df, *P* < 0.01) (Table 2), with proportionally more cells in the abdominal and buccal ganglia in adults (43% of total) compared with juveniles (26%). That result should be less sensitive to differences in the methods and suggests differential development of the number of FMRFamide-positive neurons in the different ganglia, though there could also be differential changes in the size or intensity of staining of the neurons.

**Table 2.**
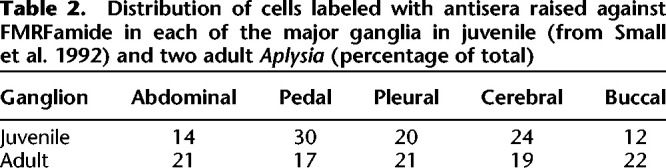
Distribution of cells labeled with antisera raised against FMRFamide in each of the major ganglia in juvenile (from Small et al. 1992) and two adult *Aplysia* (percentage of total)

There was staining for each of the five peptide neurotransmitters that we focused on in cell bodies in all of the ganglia, except that there was no staining with anti-PHI antiserum in the pedal and buccal ganglia or with anti-NPY antiserum in the buccal ganglion (Table 1). Similarly, there was staining with antiserum against 5HT in all of the ganglia except for the pleural and buccal ganglia, in agreement with Hawkins (1989). The negative result for NPY in the buccal ganglion disagrees with Jing et al. (2007), who observed a single relatively small neuron that stained with anti-NPY antiserum in each hemiganglion. We may have missed that neuron (and some other positive neurons) because we stained only alternating sections with the anti-NPY antiserum.

**Figure 3.**
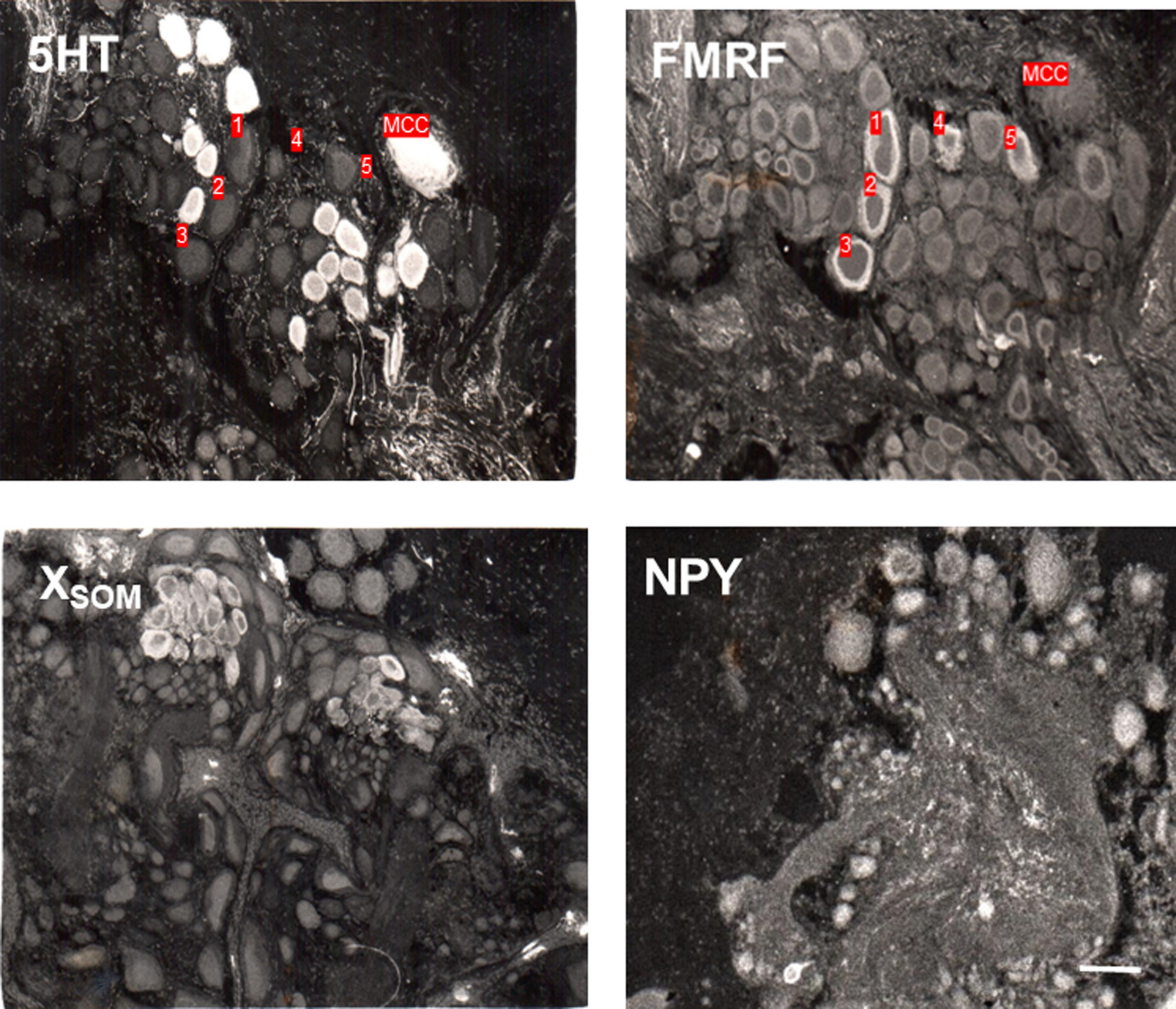
Examples of staining of the cytoplasm with antisera raised against serotonin (5HT), *Aplysia* FMRFamide, and mammalian somatostatin (SOM) and neuropeptide Y (NPY) in a region at the anterior end of the cerebral ganglion that has staining with antisera raised against many different transmitters. The anti-SOM antiserum stains an unknown peptide, X_SOM_. (MCC) Metacerebral cell, which was in the plane of section of two of the four micrographs. The numbers indicate individual neurons, none of which were double-labeled with antisera raised against 5HT and FMRFamide in adjacent sections.

**Figure 4.**
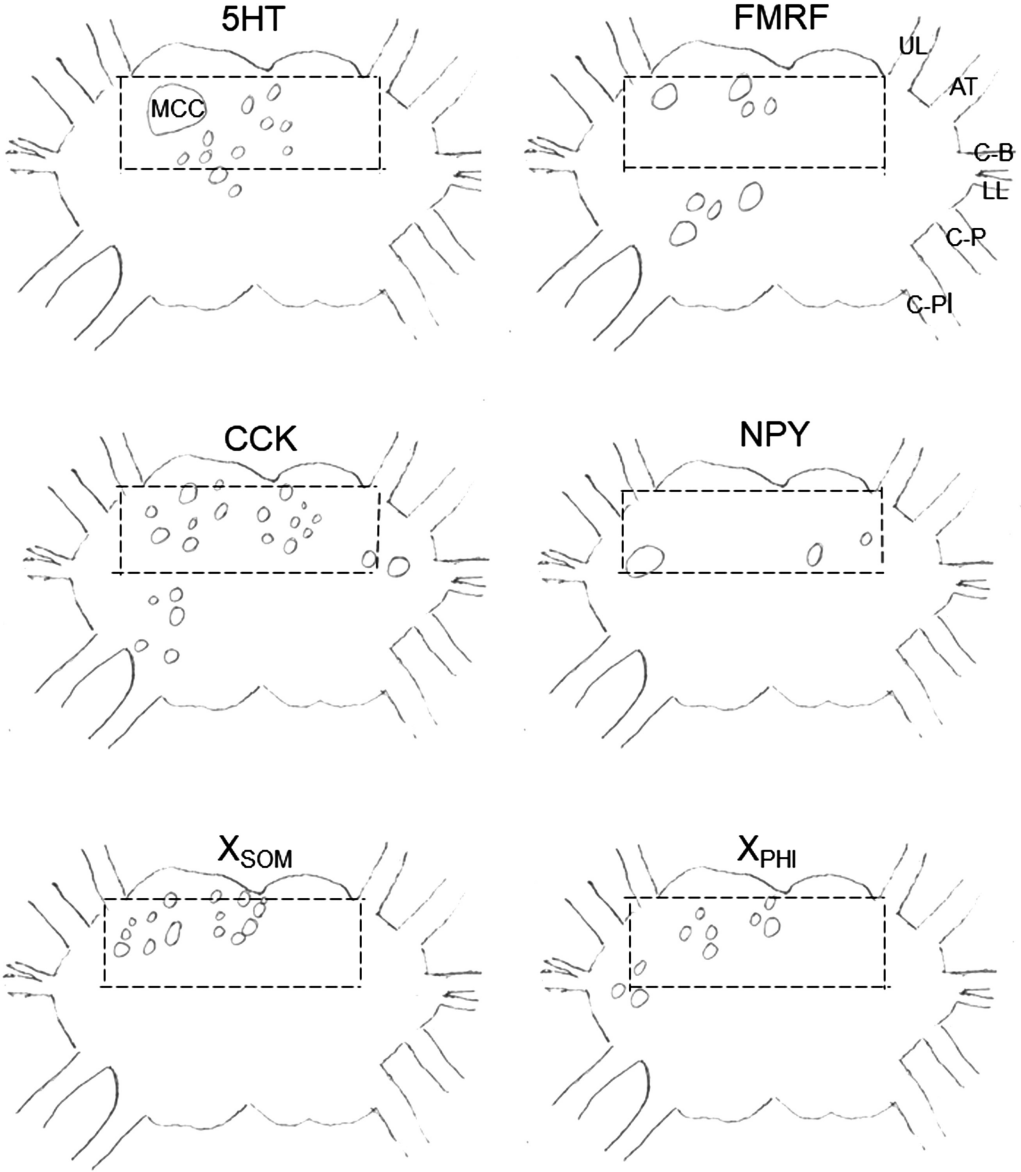
Schematic drawings illustrating the outline (dashed lines) of a region in the cerebral ganglion that has staining with antisera raised against many different transmitters, and the locations and sizes of cells that stained with antisera raised against different transmitters in that region from representative sections. The anti-SOM and ant-PHI antisera stain the unknown peptides X_SOM_ and X_PHI_, respectively. (MCC) Metacerebral cell, (C-B) cerebral–buccal connective, (C-P) cerebral–pedal connective, (C-Pl) cerebral–pleural connective, (UL) upper labial nerve, (AT) anterior tentacular nerve, (LL) lower labial nerve. (Drawing of the ganglion modified from Rosen et al. 1991.)

We observed at least some staining by antisera raised against many different modulatory transmitters in a region at the anterior end of the cerebral ganglion. Figure 3 illustrates cells with staining of the cytoplasm by antisera raised against 5HT (including the metacerebral cells), FMRFamide, mammalian SOM, and NPY in that region, only part of which is shown in the micrographs. Figure 4 shows schematic drawings outlining the entire region (dashed lines) and the locations and sizes of cells stained with antisera against those substances plus CCK and mammalian PHI in that region. There was also staining of cells with antisera raised against galanin, BPP, and enkephalin in the same region (data not shown), for a total of nine out of the 12 substances that had staining of cells anywhere. In addition, for each of the modulatory substances shown in Figure 4, there was also a higher density of stained cells in that region than in the rest of the cerebral ganglion. These results suggest that this may be a modulatory region of the nervous system.

A focus of the study was the extensive multiple staining of cell bodies with different combinations of antisera. There was double and triple staining of cell bodies with antisera against FMRFamide and CCK with each other and with each of the other peptides (Table 3; Fig. 1), including double staining of R2 with antisera against FMRFamide and CCK. There was no double staining with antiserum against 5HT and antisera against any of the peptides that we focused on, as illustrated in Figure 3 for FMRFamide. However, there was double staining with antiserum against 5HT and antisera against several peptides in our more extensive preliminary screen, including NPK, BPP, enkephalin, and CGRP. There was also double staining with additional combinations of antisera, including those against substance P and PHI, galanin and enkephalin, BPP and enkephalin, and BPP and FMRFamide.

**Table 3.**
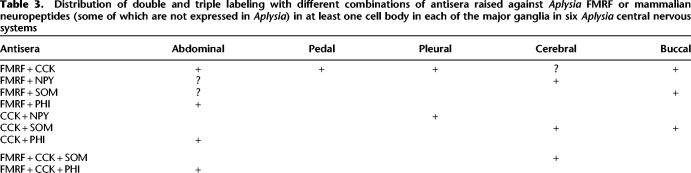
Distribution of double and triple labeling with different combinations of antisera raised against *Aplysia* FMRF or mammalian neuropeptides (some of which are not expressed in *Aplysia*) in at least one cell body in each of the major ganglia in six *Aplysia* central nervous systems

The multiple staining could be due to cross-reactivity between the antibodies. However, there was double staining for different pairs of peptides only in certain ganglia; for example, there was staining with antisera against FMRFamide and PHI individually in cells in the abdominal, pleural, and cerebral ganglia but double staining only in the abdominal ganglion. Similarly, in the example shown in Figure 1, there were individual cells that were triple-labeled with antisera against FMRFamide, CCK, and PHI in adjacent sections (for example, those labeled 2, 3, 4, and 8) and cells that were double-labeled with antisera against FMRFamide and PHI (7), but there were other cells that were labeled only with antiserum against FMRFamide (1) or CCK (5 and 6). This pattern of results would not be expected if an antibody to one of these peptides also recognized another peptide, suggesting that there was little cross-reactivity.

**Figure 5.**
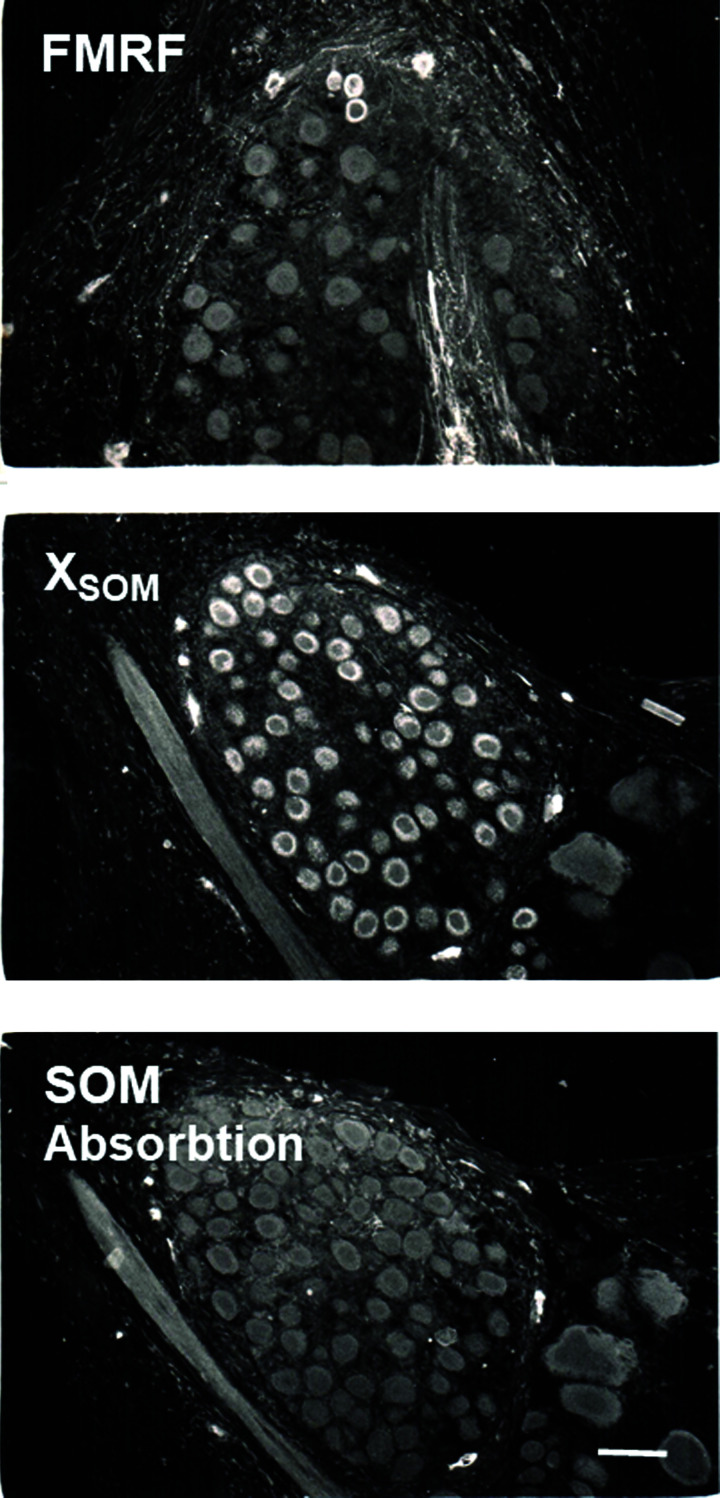
Examples of staining with antisera raised against *Aplysia* FMRFamide and mammalian SOM in the bag cell cluster of the abdominal ganglion. The anti-SOM antiserum stains an unknown peptide, X_SOM_. The staining with antiserum against SOM in an adjacent section was blocked by absorption with 10^−5^ M SOM(1–14).

There was also staining for some of these peptides in the bag cell cluster of the abdominal ganglion, which is thought to be a homogenous group of cells that produce egg-laying hormone and three bag cell peptides (Strumwasser et al. 1980; Sossin et al. 1990). Figure 5 illustrates staining of a small group of cells near the edge of the bag cell cluster with antisera against FMRFamide, as well as staining of most of the bag cells by antiserum against mammalian SOM. That staining was fully blocked by absorption with 10^−5^ M SOM(1–14) and partially blocked with 10^−6^ M SOM(1–14). These results suggest that the bag cells express a peptide that is recognized by antiserum against mammalian SOM (X_SOM_). In some cases, that peptide may be coexpressed with FMRFamide in addition to egg-laying hormone and three bag cell peptides.

## Discussion

We observed staining with antisera against FMRFamide, CCK, NPY, and mammalian SOM and PHI (for which the *Aplysia* antigens X_SOM_ and X_PHI_ are unknown), as well as the monoamine transmitter 5HT, in individual neurons in the central nervous system of adult *Aplysia*. These results suggest that these or immunogenically similar peptides might play modulatory roles in additional aspects of behavior. Our results are generally similar to those of previous studies of *Aplysia* that showed staining with antisera against FMRFamide (Brown et al. 1985; Schaefer et al. 1985; Lloyd et al. 1987; Small et al. 1992; Vilim et al. 2010), CCK (Vigna et al. 1984; Ono 1986, 1989; Soinila and Mpitsos 1991), NPY (Rajpara et al. 1992; Jing et al. 2007), and mammalian PHI (Kuramoto et al. 1985; Yui et al. 1985), though subsequent genomic studies have found that PHI is not expressed in *Aplysia*. To our knowledge, this is the first report of staining of the *Aplysia* nervous system with antiserum raised against mammalian SOM. In a preliminary screen, we also observed staining with antisera raised against several additional peptides that have been described in another gastropod mollusk, *Bulla gouldiana*, including enkephalin, CGRP, and galanin (Roberts et al. 1989).

### Distribution and colocalization of neuropeptide-immunoreactive cells

Small et al. (1992) mapped the locations of cell bodies that stained with antisera against FMRFamide in the central nervous system of juvenile *Aplysia*. We obtained a similar map in adult *Aplysia* (Fig. 2A,B) except that we observed a difference in the distribution of labeled cells in the different ganglia in juveniles compared with adults (Table 2), suggesting differential development of the number, size, or intensity of staining of neurons in the different ganglia. We also observed staining with anti-FMRFamide antiserum in the giant identified neurons R2 and LPl1, in agreement with Small et al. (1992) and Brown et al. (1985). There were cell bodies that stained with antisera raised against FMRFamide, CCK, and SOM in each of the major ganglia, but there were cell bodies that stained with antisera raised against NPY and PHI in only some of those ganglia, similar to 5HT. In addition, there were fibers that stained with antisera raised against each of the transmitters in all of the ganglia, suggesting that the peptides are transported to exert actions at the level of the nerve endings. That observation includes the abdominal and pleural ganglia, which are the locations of mechanosensory neurons that undergo 5HT- and FMRFamide-dependent synaptic plasticity during learning (Mackey et al. 1989; Small et al. 1992), suggesting that the other peptide transmitters could contribute to that plasticity as well.

There was double and triple labeling of individual neurons with all possible combinations of antisera against FMRFamide and CCK with each other and with each of the other peptides that we focused on. However, there was no double labeling of any of these peptides with antiserum against 5HT, though there was double labeling of 5HT with antisera against other peptides in our preliminary screen and also in some mammalian neurons (Hökfelt et al. 1992). There was double and triple labeling with antisera against different combinations of peptides in different ganglia and individual neurons, but other ganglia and neurons were labeled with antisera against only one of those peptides. This labeling pattern would not be expected if one antibody were recognizing two or three different peptides and suggests there was little cross-reactivity. In addition, we found that the bag cells, which are thought to be a homogenous group of cells that express three bag cell peptides and egg-laying hormone (Strumwasser et al. 1980; Sossin et al. 1990) as well as NPY (Rajpara et al. 1992), also stain with antisera raised against mammalian SOM and, in some cases, FMRFamide. These results suggest that individual bag cells may express six or seven different peptide transmitters, some of which are thought to have inhibitory actions in *Aplysia* or other species (Burgus et al. 1973; Rajpara et al. 1992; Small et al. 1992; Bruns et al. 1995).

Similarly, individual ventral neurons in the buccal ganglion stain with antisera against FMRFamide and also express small cardioactive peptides A and B (SCPA and SCPB) (Lloyd et al. 1987), and individual buccal motor neurons express SCPs, buccalins, and myomodulins as well as the conventional transmitter acetylcholine (ACh) (Cropper et al. 1991; Brezina et al. 1995; Vilim et al. 2000). Likewise, the giant neurons R2 and LPl1 stain with antisera raised against FMRFamide and CCK and also use ACh (Brown et al. 1985), and B13 in the buccal ganglion stains with antiserum against CCK and also signals via ACh (Ono 1989). Although the five peptides that we focused on did not colocalize with 5HT, several other peptides in our initial screen did. Thus, colocalization of peptide transmitters with each other and with conventional small molecule transmitters appears to be quite common in *Aplysia* and may also be common in other invertebrates (Li et al. 1999; Nusbaum et al. 2017; Nässel and Zandawala 2019) as well as vertebrates (e.g., Eriksdotter-Nilsson et al. 1987; Ceccatelli et al. 1989; Lindh et al. 1989; Hökfelt 1991; Hökfelt et al. 1992).

The colocalized transmitters can modulate different aspects of behavior, suggesting that they may not just act independently but could also interact in different combinations to produce complex functions. Thus, for example, individual neurons in the feeding system corelease a number of peptides that modulate different aspects of biting contractions, such that collectively they can produce contractions that could not be produced with a single modulator alone (for review, see Cropper et al. 2018).

### Chemical identity of neuropeptides in *Aplysia*

When using antibodies raised against vertebrate peptides on invertebrate tissues, there is a general concern regarding specificity (for discussion, see Veenstra 1988). In fact, there are distinct differences in structure already seen between peptides from different vertebrates and mammals (Pan and Kastin 2013). We and others have therefore examined the specificity of the staining with additional biochemical and genetic methods. Thus, for example, in earlier studies, the presence and identity of the *Aplysia* peptide FMRFamide were confirmed by genetic sequencing (Lehman et al. 1984; Schaefer et al. 1985; Taussig and Scheller 1986; Vilim et al. 2010; Zatylny-Gaudin and Favrel 2014) and HPLC (Lloyd et al. 1987). However, even FMRFamide staining is subject to some uncertainty, since the FMRFamide antibody also recognizes FLRFamide (Small et al. 1992).

Using a C-terminally directed CCK antiserum raised against nonsulfated porcine CCK Vigna et al. (1984) reported staining of neurons and processes in all ganglia of *Aplysia* except the pleural ganglia. Based on biochemical analyses, they concluded that the immunoreactivity represents a small peptide (eight to 17 amino acids) that is similar but not identical to mammalian gastrins and CCKs (Vigna et al. 1984). Gastrin and CCK share the same five C-terminal amino acids (Rehfeld et al. 2007), and the precursor of CCK was confirmed by genetic sequencing in *Aplysia* (Zatylny-Gaudin and Favrel 2014).

Rajpara et al. (1992) have reported staining with antisera against NPY in bag cells. Chemical analysis using mass spectrometry and genetic sequencing indicates that the peptide aplysia(ap)NPY is an amidated, 40-amino-acid-long peptide that is highly homologous to mammalian NPY (Rajpara et al. 1992; Jing et al. 2007).

Two early studies reported staining with antisera against mammalian PHI in *Aplysia* (Kuramoto et al. 1985; Yui et al. 1985), but the presence of PHI in *Aplysia* has not been confirmed with more recent genomic, transcriptomic, or evolutionary analyses (Jékely 2013; Jiang et al. 2022; Orvis et al. 2022). We therefore refer to this peptide as X_PHI_.

To our knowledge, this is the first report of staining with antisera raised against mammalian SOM in the *Aplysia* nervous system, but the true identity of the *Aplysia* antigen remains to be revealed. There was staining in most or all of the bag cells, which express three bag cell peptides and egg-laying hormone (Strumwasser et al. 1980; Sossin et al. 1990) as well as NPY (Rajpara et al. 1992), but none of those has obvious sequence similarity to SOM. Absorption with mammalian SOM(1–14) peptide blocks the staining, suggesting that the antiserum raised against mammalian SOM(1–14) recognizes an *Aplysia* peptide (X_SOM_) that has some amino acid sequences in common with mammalian SOM (Fig. 5). We attempted to characterize both SOM and galanin immunoreactivities using immunochemical methods, including gel filtration chromatography and liquid chromatography–mass spectrometry, but were unable to identify the sequence of amino acids present in SOM(1–14) or in galanin(1–15) in the relevant gel filtration chromatography fractions. This failure might be due to very low concentrations of the peptides or technical issues. However, early and more recent studies based on genomic, transcriptomic, and evolutionary analyses have shown that genuine mammalian SOM is not expressed in *Aplysia* (Jékely 2013; Jiang et al. 2022; Orvis et al. 2022). More generally, neuropeptides recognized in *Aplysia* by antisera raised against mammalian or vertebrate peptides may not be the same as their vertebrate counterparts, though they could be relatives. Thus, the SOM antibody may recognize a molluskan ortholog, allatostatin C (Veenstra 2010; Jiang et al. 2022). SOM also has structural similarities to urotensin II (Tostivint et al. 2008), whose gene is expressed in *Aplysia* (Romanova et al. 2012).

### Functions of neuropeptides in *Aplysia*

The biochemical studies have been further validated by functional experiments. FMRFamide has been extensively studied in *Aplysia* and has been shown, for example, to mimic the inhibitory effects of tail shock on the sensory neurons (Small et al. 1992). The cholinergic neuron B13 in buccal ganglion stains with antisera against CCK (Ono 1989). When B13 neurons are activated and combined with cholinergic antagonists, a slow depolarizing response still remains in the follower neurons (B3 and B40), but an involvement of the excitatory peptides CCK or gastrin could not be demonstrated (Ono 1989). However, Hammond et al. (1987) have shown that CCK-8 induces a decrease in Ca^2+^ in snail neurons. apNPY mimics the long-lasting, prolonged inhibition produced by bag cells (Rajpara et al. 1992), which may therefore express and release two inhibitory peptide transmitters, including FMRFamide and three or four excitatory peptides, all of which could interact in their signaling. The simple nervous system of *Aplysia* is advantageous for further testing the behavioral functions of these peptides individually and in coreleased combinations.

## Material and Methods

*Aplysia californica* (50–150 g; obtained from Pacific Bio-Marine Supply Co.) were maintained at 15°C in circulating Instant Ocean. The animals were anesthetized by injection of isotonic MgCl_2_, and the central nervous system was removed. Sections (10–12 µm thick) were cut in a cryostat at −20°C, mounted onto gelatin-coated glass slides, and processed for immunocytochemistry (Kistler et al. 1985). Briefly, alternating sections were incubated with polyclonal rabbit antibodies to (1) FMFRamide (from Immuno Nuclear), (2) the unsulfated CCK octapeptide (described in Frey 1983), (3) PHI(27) (Fahrenkrug and Pedersen 1984), (4) SOM(15–28) (Elde et al. 1978), or (5) polyclonal goat antibodies to NPY (personal gift from Thue Schwartz) and (6) guinea pig antibodies to 5HT (Steinbusch et al. 1983) in a humid chamber overnight at 4°C. Following several rinses with PBS, the sections were incubated for 30 min at 37°C with fluorescein isothiocyanate (FITC)-conjugated secondary antirabbit antibodies raised in goat (for CCK, FMRFamide, PHI, and SOM antibodies; from Boehringer Mannheim), antigoat antibodies raised in donkey (for NPY antibody; from Nordic Biosite) and anti-guinea pig antibodies raised in swine (for 5HT; from Nordic Biosite). Dilutions of primary and secondary antibodies are shown in Table 4. The sections were then rinsed in PBS, coverslipped in a glycerol:PBS (3:1) solution containing 0.1% *p*-phenylene-diamine, examined in a Zeiss standard fluorescence microscope equipped with an oil darkfield condenser and proper filter combinations for FITC fluorescence, and photographed.

**Table 4.**
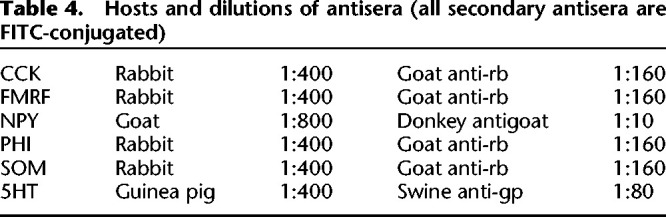
Hosts and dilutions of antisera (all secondary antisera are FITC-conjugated)

### Competing interest statement

T.H. has shares in Lundbeck and Bioarctic. The other authors declare no competing interests.
